# Optimal dynamic soaring trades off energy harvest and directional flight

**DOI:** 10.1016/j.isci.2025.112540

**Published:** 2025-04-28

**Authors:** Lunbing Chen, Yufei Yin, Yang Xiang, Suyang Qin, Hong Liu

**Affiliations:** 1 J. C. Wu Center for Aerodynamics, School of Aeronautics and Astronautics, Shanghai Jiao Tong University, Shanghai 200240, China

**Keywords:** Evolutionary ecology, Energy modeling

## Abstract

Wandering albatrosses use dynamic soaring to achieve low-cost, continuous flight over thousands of kilometers. While previous research has primarily focused on energy harvest, directional flight may be more important for reaching a destination. Through numerical simulations validated by flight tracking data, this study reveals a trade-off between maximizing energy harvest and achieving the fastest directional progress: maximizing energy gain increases mechanical energy but slows target-oriented movement, while prioritizing directional flight reduces energy gain. Albatrosses balance this trade-off through a step-selection strategy, dividing each flight cycle into energy-harvest and directional-flight phases, each with distinct priorities. The duration of each phase is influenced by environmental shear strength: low-shear conditions allocate more time to harvest energy, while high-shear conditions favor faster directional movement. By optimizing this balance, albatrosses achieve efficient destination-oriented soaring. These insights enhance our understanding of pelagic bird flight and could inspire high-efficiency robotic albatrosses.

## Highlights

• Introduce directional flight as equally important as energy harvest in dynamic soaring
• Reveal conflicting flight trajectories between directional flight and energy harvest
• Show that albatrosses resolve this trade-off using a step-selection strategy
• Analyze real bird tracking data to evaluate actual flight behavior

## Graphical abstract

**Figure:**
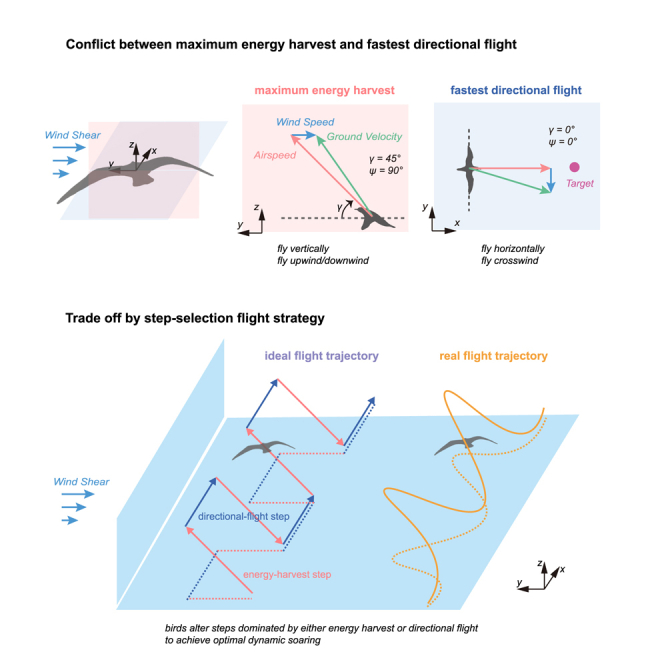

## Introduction

Compared to birds such as wandering albatrosses,^1^^,^^2^^,^^3^ frigatebirds,^4^ and shearwaters^5^—capable of covering thousands of kilometers over several months—unmanned aerial vehicles (UAVs) consume significantly more energy while achieving limited ranges of only a few kilometers or hours of flight.^6^^,^^7^ One major reason for this disparity lies in the birds’ extraordinary ability to harness environmental energy,^6^^,^^8^^,^^9^ with dynamic soaring being among the most effective methods.^1^^,^^2^^,^^3^^,^^4^^,^^5^ Through dynamic soaring, birds can migrate non-stop at minimal energy cost,^1^^,^^2^^,^^3^^,^^4^^,^^5^ maintaining flight toward their target.

Energy harvest is widely acknowledged as a core aspect of dynamic soaring, determined primarily by two factors: the bird’s flight trajectory and the environmental wind field. A bird’s flight trajectory typically follows a periodic cycle,^3^^,^^10^^,^^11^^,^^12^ where it ascends into the wind to a maximum altitude before descending in a turn toward the ground or water.^3^^,^^10^ The critical feature of the wind field lies in spatiotemporal wind gradients.^5^^,^^10^^,^^12^ In 1883, Rayleigh first explained this energy harvest process, linking the bird’s trajectory with the wind field: during ascent, the bird encounters increasing headwinds as it moves from slower to faster air, while descent weakens the tailwinds as it moves from faster to slower air.^11^ Both processes amplify the bird’s airspeed and airspeed-relative kinetic energy. Rayleigh’s theory has gained widespread acceptance,^5^^,^^6^^,^^10^^,^^12^ with Taylor et al. summarizing it succinctly as “descend downwind; ascend upwind”.^12^

However, this conventional understanding does not fully explain how birds achieve directional flight toward specific targets while using dynamic soaring. Flight is not solely about energy efficiency; natural selection suggests that birds must balance factors such as time, risk, safety, and other trade-offs.^13^ For example, during home-range movements or outbound flights, birds often prioritize faster access to resources over energy conservation.^14^^,^^15^ Therefore, dynamic soaring must account for directional movement, integrating how birds balance competing needs like energy efficiency and travel speed to achieve optimal outcomes.

This paper distills the dynamics of dynamic soaring into two fundamental aspects: energy harvest and directional flight. Energy harvest involves maximizing energy acquisition from the environment, while directional flight focuses on minimizing time to reach a destination. The central aim of this study is to explore how birds balance these objectives to achieve optimal flight in one dynamic soaring cycle, using numerical methods and validated by experimental tracking data. Although birds can achieve energy-efficient flight in various directions, crosswind flight—broadly defined here as travel directions between 60° and 120° relative to the wind direction—remains the dominant mode of directional flight^1^^,^^15^^,^^16^^,^^17^ and serves as the primary framework in this paper for examining the trade-offs inherent in dynamic soaring.

## Results

### Modeling of dynamic soaring

**Figure 1:**
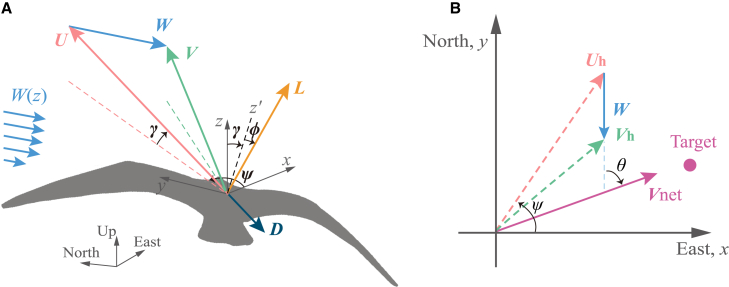
3-degree-of-freedom model of glider flight in wind shear (A) 3D schematic diagram. (B) Projection onto the north-east plane. The inertial reference frame where the x, y, and z positions correspond to the East, North, and Up coordinates. U, V, and W correspond to the airspeed, ground velocity and wind velocity. γ is the air-relative pitch angle defined as the angle between U and the horizontal plane, signed positive when climbing and negative when diving. ψ is the air-relative yaw angle between the positive x-direction and the horizontal component of the airspeed vector $U_{h}$, signed positive when y>0 and negative when y<0. ϕ is the roll angle. The positive directions of γ, ψ, and ϕ, are defined based on the standard aircraft principal axes system.^20^L and D are the lift and drag force, respectively. W(z) is the wind profile. The colorful dashed lines represent vector projections into the East-North plane (xy-plane), with the target referring to a specific point within that plane. $V_{h}$ represents the horizontal components of ground velocity. $V_{\text{net}}$ is the horizontal traveling net speed along the travel direction and θ is the travel direction defined by the angle between the wind direction and $V_{\text{net}}$.

The point-mass 3-degree-of-freedom glider model in a logarithmic shear wind profile has been widely accepted for the study of dynamic soaring.^16^^,^^18^^,^^19^ Figure 1 shows the definition sketch of the key vectors (U, V, W, $V_{\text{net}}$, L, D), scalars ($C_{L}$, $C_{D}$) and the axis system (x, y, z, γ, ψ, ϕ, θ) used in the modeling. This study emphasizes travel direction θ as a fundamental premise. Specifically, travel direction is defined relative to wind direction, and the analysis is confined to microscale dynamic soaring within a single cycle, where the energy harvested from albatross-air interactions is highlighted.^17^ The angle θ is set between 60° and 120°, consistent with observed angles in real microscale flight: approximately 55° for homing and 80° for non-homing albatrosses (Figure 4D in Goto et al.^17^), supporting the validity of this range.

The equations of motion (EOMs) of this glider model can be derived as:

(Equation 1) $$\begin{array}{c} m\dot{U}=-D-mg\;\sin\;\gamma +m\sigma \dot{z}\cos\;\gamma \;\sin\;\psi \end{array}$$

(Equation 2) $$\begin{array}{c} mU\dot{\gamma }=L\;\cos\;\phi -mg\;\cos\;\gamma -m\sigma \dot{z}\sin\;\gamma \;\sin\;\psi \end{array}$$

(Equation 3) $$\begin{array}{c} mV\dot{\psi }\cos\;\gamma =L\;\sin\;\phi +m\sigma \dot{z}\cos\;\psi \end{array}$$

(Equation 4) $$\begin{array}{c} \dot{x}=U\;\cos\;\gamma \;\cos\;\psi \end{array}$$

(Equation 5) $$\begin{array}{c} \dot{y}=U\;\cos\;\gamma \;\sin\;\psi -W \end{array}$$

(Equation 6) $$\begin{array}{c} \dot{z}=U\;\sin\;\gamma \end{array}$$

where the dots represent time derivation such as $\dot{U}=dU/dt$. m is the glider mass and g is the acceleration due to gravity. Following the classical steady aerodynamics, lift and drag are specified according to $L=1/2C_{L}\rho SU^{2}$ and $D=1/2C_{D}\rho SU^{2}$, where, S is the wing area and ρ is the air density. The drag coefficient is $C_{D}=C_{D0}+C_{L}^{2}/\pi \lambda$ according to Prandtl’s classical lifting-line theory^21^ with the lift coefficient $C_{L}$, the zero-lift drag coefficient $C_{D0}$ and the aspect ratio λ.

The wind is assumed to be blowing from north to south when W>0 and the wind profile can be modeled using the logarithmic model, i.e.,

(Equation 7) $$\begin{array}{c} W\left(z\right)=W_{\text{ref}}\frac{\log\left(z/h_{0}\right)}{\log\left(h_{\text{ref}}/h_{0}\right)} \end{array}$$

where $W_{\text{ref}}$ is the reference wind speed, which can vary depending on the computational requirements, $h_{\text{ref}}=10m$ is the height at which the reference wind speed is specified, and $h_{0}=0.03m$ is the surface roughness length. The corresponding wind shear strength is defined as,

(Equation 8) $$\begin{array}{c} \sigma \left(z\right)=\frac{dW}{dz}=\frac{W_{\text{ref}}}{\log\left(h_{\text{ref}}/h_{0}\right)}\frac{1}{z} \end{array}$$

For the near-surface atmospheric boundary layer, wind speed increases with height above the surface, so, σ is always positive. The mean shear strength denoted as $\bar{\sigma }$, can be expressed as,

(Equation 9) $$\begin{array}{c} \bar{\sigma }=\frac{1}{h_{\text{ref}}-h_{0}}\int_{h_{0}}^{h_{\text{ref}}}\sigma dz=\frac{W_{\text{ref}}}{h_{\text{ref}}-h_{0}} \end{array}$$

which represents the shear strength in a generalized sense.

### Energetics of dynamic soaring

The mechanical energy of the albatross is the sum of gravitational potential energy and aerodynamic kinetic energy,^5^^,^^10^^,^^18^ expressed as, $E=mgz+1/2mU^{2}$. The rate of change of mechanical energy is given by the time derivative of E, as follows, $\dot{E}=mg\dot{z}+mU\dot{U}$. Substituting the expressions for $\dot{z}$ and $\dot{U}$ from Equation 1, we obtain:

(Equation 10) $$\begin{array}{c} \dot{E}=\dot{E_{1}}+\dot{E_{2}}=\frac{1}{2}U^{2}m\sigma \;\sin\;2\;\gamma \;\sin\;\psi -DU\text{.} \end{array}$$

Since the second term ($\dot{E_{2}}=-DU$) is always negative, the rate of energy harvest depends on the first term ($\dot{E_{1}}$). $\dot{E_{1}}$ first highlights that dynamic soaring, as an energy-harvest strategy, exploits spatial gradients in horizontally moving air (wind shear, σ), where wind velocity changes with altitude. If birds were flying in a uniform flow, Galilean invariance suggests they could remain airborne even in still air. However, air resistance in still air makes sustained flight without wing flapping impossible, thus necessitating wind shear for dynamic soaring. Therefore, dynamic soaring is not limited to the atmospheric boundary layer; any flow structure capable of generating shear flows, such as wind-wave interactions and wind-topography interactions, can also serve as an energy source.^9^ $\dot{E_{1}}$ also shows that energy harvest is maximized when flying in the direction that optimizes the energy harvest coefficient,

(Equation 11) $$\begin{array}{c} \eta =\sin\;2\;\gamma \;\sin\;\psi \text{.} \end{array}$$

Energy is harvested from the wind shear when η>0 and lost when η<0, at a rate proportional to ‖η‖. The cycle-averaged energy harvest coefficient,

(Equation 12) $$\begin{array}{c} \bar{\eta }=\frac{1}{T}\int_{0}^{T}\eta dt=\frac{1}{T}\int_{0}^{T}\sin\;2\;\gamma \;\cos\;\psi dt\text{,} \end{array}$$

signifies the birds’ inclination toward energy harvest since γ and ψ are birds’ state parameters, with a larger value indicating a stronger preference for energy harvest.

### Directional flight of dynamic soaring

For directional flight shown in Figure 1B, the net traveling speed can be derived as,

(Equation 13) $$\begin{array}{c} V_{\text{net}}=U\;\cos\;\gamma \;\sin\left(\psi +\theta \right)-W\;\cos\;\theta \text{.} \end{array}$$

Since the second term is deterministic for the known wind condition and known travel direction, the instantaneous directional travel is always maximized by flying in an instantaneous direction that maximizes the dimensionless quantity, defined as the directional flight coefficient,

(Equation 14) $$\begin{array}{c} \epsilon =\cos\;\gamma \;\sin\left(\psi +\theta \right)\text{.} \end{array}$$

So, the bird flies toward the travel direction when ϵ>0 and deviates from the travel direction when ϵ<0, at a rate proportional to ‖ϵ‖. The cycle-averaged directional flight coefficient,

(Equation 15) $$\begin{array}{c} \bar{\epsilon }=\frac{1}{T}\int_{0}^{T}\epsilon dt=\frac{1}{T}\int_{0}^{T}\cos\;\gamma \;\sin\left(\psi +\theta \right)dt\text{,} \end{array}$$

reflects the bird’s propensity for directional flight, with a larger value indicating a stronger preference for directional flight. The cycle-averaged definitions offer descriptions of the overall process for directional flight.

### Numerical optimization

The numerical optimization in this study is to find the state and control time histories that maximize the objective function (the cycle-averaged horizontal traveling net speed ${\bar{V}}_{\text{net}}$) while satisfying the special EOMs (Equation 1) and additional constraints, that is,

(Equation 16) $$\max.:{\bar{V}}_{\text{net}}\left(\tilde{s},\tilde{u}\right)$$

(Equation 17) $$w.r.t.:\tilde{s},\tilde{u}$$

(Equation 18) $$s.t.:C\left(\tilde{s},\tilde{u}\right)$$

${\bar{V}}_{\text{net}}=\sqrt{{\Delta x}^{2}+{\Delta y}^{2}}/T$, where, Δx and Δy denote the x and y coordinate distances between the starting point and the endpoint in one cycle, and T represents the duration. $\tilde{s}=\left(U,\psi ,\gamma ,x,y,z\right)$ is the glider’s state, $\tilde{u}=\left(C_{L},\phi \right)$ is the control input, and C is the set of constraints (for more details on the constraints, please refer to the section “constraints of the optimization problem”). Other details on the procedure can be found in the work of Sachs et al.,^22^ Bousquet et al.,^16^ and Bower.^18^ IPOPT is utilized for current optimization.^23^

**Figure 2:**
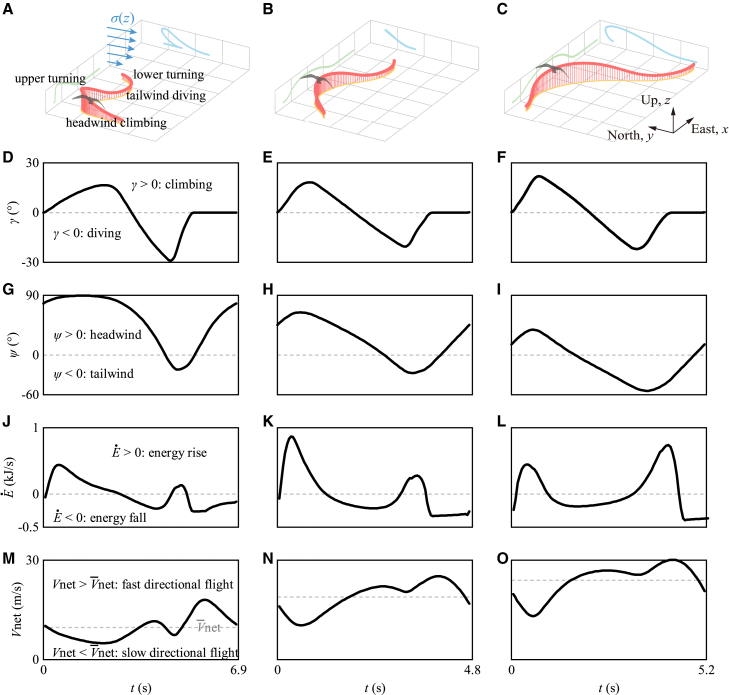
Dynamic soaring through climbing-diving cycles to trade energy for directional flight During energy rise phases, birds harvest energy from the environment, while in energy fall phases, they allocate it to maintain directional flight. (A–C) Show typical trajectories (light red lines represent 3D trajectories) and their projections (light yellow, light green, and light blue solid lines represent 2D projections in the xy, xz, and yz planes, respectively). (D–O) Depict the key quantities — air-relative pitch angle (γ), yaw angle (ψ), mechanical energy change rate ($\dot{E}$), and net traveling speed ($V_{\text{net}}$). The first to third columns of figures correspond to flight directions θ=60°, 90°, and 120°. Simulations are performed with the objective of maximizing ${\bar{V}}_{\text{net}}$ at a reference wind speed ($W_{\text{ref}}$) of 10m/s. Supplementary results regarding airspeed U and lift coefficient $C_{L}$ are provided in Figure S5 in the supplemental information.

The optimization algorithm has been validated in terms of flight trajectories and minimum reference wind speeds as detailed in the section “model validation”. Additionally, beyond the logarithmic wind model, the robustness of the conclusions has also been verified under the sigmoid wind model. Further details on this validation can be found in the section “influence of Sigmoid wind model”. In the main text, the typical trajectories depicted in Figure 2A have also been validated as correct. This trajectory closely resembles the experimental observations^10^^,^^24^^,^^25^ and is consistent with existing numerical simulation results.^18^

### Data analysis from the literature

There have been numerous experimental observations regarding the dynamic soaring of wandering albatrosses. For example, Sachs et al. tracked a short (3 km) soaring process with a high (10 Hz) sampling rate,^10^ and Yonehara et al. did long (order of 1000 km) tracks sampled at a lower rate (1 Hz).^24^ The analysis in this study is based on the data provided by Yonehara et al.,^24^ which includes flight trajectory latitude and longitude data totaling 185 h of flight for a total of four wandering albatrosses. The tracked albatrosses were engaged in prey-capture events,^24^^,^^26^ a purposeful behavior, leading us to believe that the analysis of these data can effectively reflect the relationship between energy harvest and directional flight.

The challenge lies in determining $\bar{\sigma }$, $\bar{\eta }$, and $\bar{\epsilon }$ from the latitude and longitude data (x and y coordinates). Based on Equation 9, assuming constant values of $h_{\text{ref}}=10m$ and $h_{0}=0.03m$, determining $\bar{\sigma }$ requires finding the value of $W_{\text{ref}}$. Due to the difficulty in segmenting actual flight data for each cycle, instead of averaging over individual cycles, T=300s is chosen as a trade-off between obtaining enough data points to accurately estimate wind conditions while maintaining high temporal resolution.^24^ Also, according to Equations 12 and 15, determining $\bar{\eta }$ and $\bar{\epsilon }$ involves obtaining the values for γ, ψ and θ.

Using the x and y coordinates, we can determine the horizontal ground velocity’s magnitude $V_{h}$ and direction $\theta_{V}\in [0{}^{\circ},360{}^{\circ})$ from the time differences in position:

(Equation 19) $$\begin{array}{c} V_{x}=\frac{\delta x}{\delta t}\text{,} \end{array}$$

(Equation 20) $$\begin{array}{c} V_{y}=\frac{\delta y}{\delta t}\text{,} \end{array}$$

(Equation 21) $$\begin{array}{c} V_{h}=\sqrt{V_{x}^{2}+V_{y}^{2}}\text{,} \end{array}$$

(Equation 22) $$\begin{array}{c} \theta_{V}=\tan^{-1}\frac{V_{x}}{V_{y}}\text{,} \end{array}$$

where δx and δy represent the distance changes during δt=1s. Please note that this calculation only accounts for the horizontal components, lacking the vertical velocity component. Referring to the work by Yonehara et al.^24^ and Shimatani et al.,^27^ a sinusoidal curve, with coefficients, ${\bar{V}}_{h}$, a and b, was fitted against the relationship $V_{h}$ and $\theta_{V}$ for each 300-s section using the following equation to obtain the wind speed magnitude and wind direction during each segment,

(Equation 23) $$\begin{array}{c} V_{h}={\bar{V}}_{h}+a\;\sin\;\theta_{V}+b\;\cos\;\theta_{V}\text{.} \end{array}$$

By assuming that the ground speed is only affected by the wind speed along flight direction, the ground speed would be maximized in pure tailwind (the wind velocity vector W and the ground velocity vector V are aligned in the same direction on the horizontal plane) and equal the sum of airspeed and wind speed, whereas it would be minimized in pure headwind (the wind velocity vector W and the ground velocity vector V are aligned in the opposite direction on the horizontal plane) and equal the wind speed subtracted from airspeed. Therefore, wind speed ($W_{0}$) is calculated as one-half of the difference between the maximum (${V_{h}}_{\max}$) and the minimum (${V_{h}}_{\min}$) values of the fitted sinusoidal curve,

(Equation 24) $$\begin{array}{c} W_{0}=\frac{{V_{h}}_{\max}-{V_{h}}_{\min}}{2}\text{.} \end{array}$$

The wind direction is determined as $\theta_{W}$ corresponding to the direction at the maximum flight speed (${V_{h}}_{\max}$) of the fitted sinusoidal curve.^24^ Since this wind speed ($W_{0}$) is usually underestimated,^24^ so further correction to the wind speed magnitude ($W_{\text{ref}}$) at $h_{\text{ref}}=10m/s$ is carried out based on a linear relationship,^24^

(Equation 25) $$\begin{array}{c} W_{\text{ref}}=1.80W_{0}-1.42\text{.} \end{array}$$

From this, $\bar{\sigma }$ can be obtained. Additionally, based on the trigonometric relationship between $W_{\text{ref}}$ and $V_{h}$, the air-relative yaw angle ψ can be calculated as,

(Equation 26) $$\begin{array}{c} U_{h}=\sqrt{V_{h}^{2}+W_{\text{ref}}^{2}-2V_{h}W_{\text{ref}}\;\cos\left(\theta_{V}-\theta_{W}\right)}\text{,} \end{array}$$

(Equation 27) $$\begin{array}{c} \psi =\sin^{-1}\left(\frac{V_{h}}{U_{h}}\sin\left(\theta_{V}-\theta_{W}\right)\right)\text{.} \end{array}$$

Due to the absence of altitude information in Yonehara et al.’s data,^24^ we cannot directly calculate γ. Instead, we utilize the observed similarity between the trends of γ and ψ, where γ increases with ψ and decreases as ψ decreases (see Figure 2). A linear fit to the numerical results at $W_{\text{ref}}=10\;m/s$ yields the equation:

(Equation 28) $$\begin{array}{c} \gamma =l\left(\theta \right)\psi -m\left(\theta \right) \end{array}$$

where,

(Equation 29) $$\begin{array}{c} l\left(\theta \right)=0.00309*\theta +0.02\text{,} \end{array}$$

(Equation 30) $$\begin{array}{c} m\left(\theta \right)=0.00427*\theta -0.47\text{,} \end{array}$$

and θ is calculated as $\left|\theta_{V}-\theta_{W}\right|$. Additionally, since our discussion focuses on crosswind dynamic soaring within the range of θ∈[60°,120°], any calculated θ values outside this range are excluded during the empirical data processing. Finally, we can estimate γ from ψ and subsequently calculate the corresponding $\bar{\eta }$ and $\bar{\epsilon }$.

## Discussion

### Dynamic soaring trading energy by climbing-diving cycles to achieve directional flight

Birds’ flight trajectories during dynamic soaring exhibit distinct three-dimensional climbing-diving cycles. As shown in Figures 2A–2C for travel directions θ=60°, 90°, and 120°, each cycle includes four phases: (1) headwind climbing, (2) upper turning, (3) tailwind diving, and (4) lower turning. Air-relative pitch angle γ and yaw angle ψ provide further kinematic details. Comparing Figures 2D–2I, the signs of γ and ψ generally align: γ>0 with ψ>0, and γ<0 with ψ<0. Both angles follow similar trends—γ increases as ψ increases and decreases as ψ decreases. Since γ reflects climbing or diving and ψ represents headwind or tailwind, these patterns suggest that climbing coincides with headwinds, and diving aligns with tailwinds.

These kinematic variations reflect the birds’ pursuit of energy harvest. When γ∈[0°,45°] and ψ∈[0°,90°] are both increasing, the birds climb toward the headwind, leading to a significant increase in the magnitude of the air-relative mechanical energy change rate ($\dot{E}$), as shown in Equation 11. Similarly, when γ∈[−45°,0°) and ψ∈[−90°,0°) are both decreasing, as the birds dive downwind, the magnitude of $\dot{E}$ also increases. These energy gains are illustrated in Figures 2J–2L, demonstrating the relationship: “ascend upwind; descend downwind”.^12^

However, energy gain is not constant throughout the cycle, as shown in Figures 2J–2L. During the transitions between positive and negative γ values— when birds are in the upper and lower turning regions— the signs of γ and ψ oppose each other, resulting in a negative rate of mechanical energy change and energy fall.

Energy fall occurs when birds use the harvested energy for directional flight. Comparing Figures 2J–2O shows that when the mechanical energy change rate ($\dot{E}$) is negative, the net traveling speed ($V_{\text{net}}$) exceeds the average, and as $\dot{E}$ reaches its minimum, $V_{\text{net}}$ peaks. This suggests that directional flight is most pronounced during the energy fall phase, with the fastest speeds occurring when energy fall is greatest.

Thus, dynamic soaring relies on trading energy to achieve directional flight, regardless of travel direction. Birds cannot always harvest energy at every point in their trajectory; instead, there are phases of energy fall. During energy rise phases, birds harvest energy from the environment, while during energy fall phases, they allocate this energy to maintain directional flight. As long as the overall energy balance remains neutral, birds can sustain continuous flight toward their travel direction through dynamic soaring.

### Step-selection flight strategy for handling the conflict between energy harvest and directional flight

**Figure 3:**
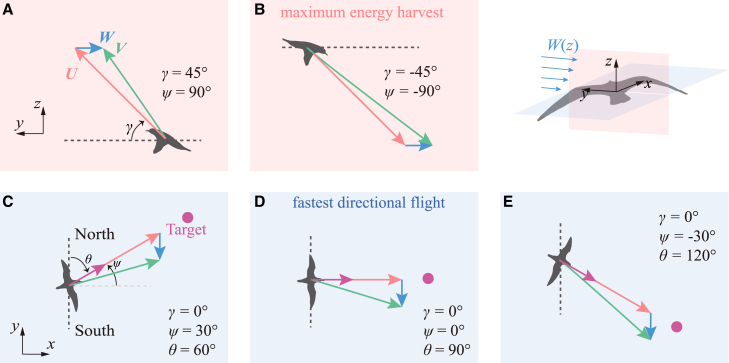
Conflict between energy harvest and directional flight Maximum energy harvest requires birds to ascend into headwinds or descend with tailwinds in a vertical plane (A and B), while achieving the fastest directional flight implies flying crosswind in a horizontal plane (C–E). Note: The vectors indicate directional relationships only, not relative speed magnitudes.

In fact, during dynamic soaring, a conflict arises between energy harvest and directional flight for birds. According to the energy harvest coefficient, η=sin2γsinψ, maximum energy harvest ($\eta_{\max}=1$) occurs when γ=45° and ψ=90° during climbing or γ=−45° and ψ=−90° during diving. This means that birds harvest the most energy when flying directly into the wind during climbing and with the wind during diving in a vertical plane, as shown in Figures 3A and 3B. Conversely, for the fastest directional flight, the directional flight coefficient, ϵ=cosγsin(ψ+θ), is maximized ($\epsilon_{\max}=1$) when γ=0° and ψ=90°−θ, indicating that the airspeed should be aligned with the flight direction in the horizontal plane. The velocity diagrams for the fastest directional flight are illustrated in Figures 3C–3E for different travel directions.

Thus, two key contradictions exist between energy harvest and directional flight. First, maximizing energy harvest requires movement in the vertical plane, while the fastest directional flight occurs in the horizontal plane. Second, maximum energy harvest depends on wind direction (either with or against it), while the fastest directional flight is determined by the travel direction. As wind and travel directions often misalign, especially during crosswind flight, the greater the disparity between them, the more pronounced the conflict becomes, particularly when θ=90°.

**Figure 4:**
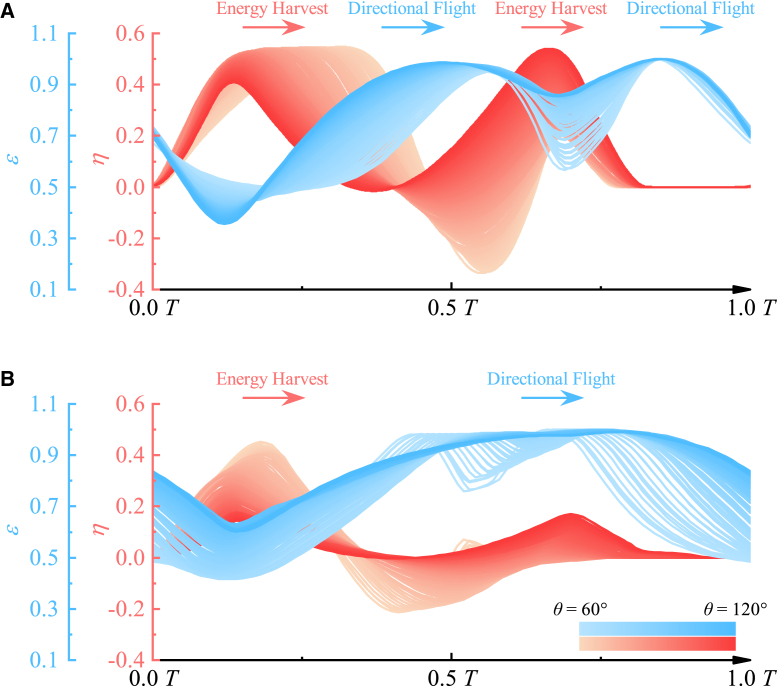
The step-selection flight strategy for dynamic soaring Energy harvest coefficient η (light red) and directional flight coefficient ϵ (light blue) change with time in one cycle. η and ϵ alternately exceed each other, implying birds alter small intervals dominated by either energy harvest or directional flight to resolve conflicts between energy harvest and directional flight. (A) for $W_{\text{ref}}=10\;m/s$ and (B) for $W_{\text{ref}}=20\;m/s$. The travel direction ranges from θ=60° to θ=120°, with curves plotted at 1° intervals.

So, how do birds manage the conflict between energy harvest and directional flight during dynamic soaring? The variations in the energy harvest coefficient (η) and directional flight coefficient (ϵ) from numerical simulations are illustrated in Figure 4. The results reveal that η and ϵ exhibit inverse trends: as η increases, ϵ decreases, and vice versa, highlighting the inherent conflict in practical scenarios.

However, the alternating peaks of η and ϵ in Figure 4 also suggest a potential solution for birds. They employ a step-selection decision-making process in real flight. In Figure 4A, within one cycle, η initially exceeds ϵ, then becomes smaller than ϵ, later exceeds ϵ again, and finally becomes smaller than ϵ once more. This shows that energy harvest and directional flight alternate as primary requirements during specific flight steps. Birds first harvest energy, then expend energy to maintain directional flight, repeating this process for continuous flight.

This process superficially resembles static soaring,^6^^,^^9^ where birds utilize thermal^28^ or orographic updrafts^29^ to gain gravitational potential energy and then glide to achieve directional flight. However, there are five critical differences. First, static soaring typically involves circling flight, resulting in minimal forward progress during energy harvest, while dynamic soaring allows birds to advance toward the travel direction at the same time (ϵ remains positive in Figure 4). Second, the timescale for completing a flight cycle in static soaring is typically on the order of $O\left(10^{3}\right)$ seconds,^4^^,^^28^ while in dynamic soaring, it is about O(1) second. Third, static soaring compensates for drag losses by harvesting gravitational energy from rising air currents, whereas dynamic soaring harvests aerodynamic kinetic energy from spatiotemporal wind gradients.^12^ Fourth, the energy exchange in one static soaring cycle is on the order of $O\left(10^{3}\right)\;J/\text{kg}$ (with altitude changes of $O\left(10^{2}\right)\;m$),^4^^,^^28^ while in dynamic soaring, it is on the order of $O\left(10^{2}\right)\;J/\text{kg}$ (with velocity changes of O(10)m/s).^17^^,^^26^ Finally, considering both the energy exchange and the time required to complete one cycle, the energy harvest rate of dynamic soaring is approximately two orders of magnitude greater than that of static soaring, which highlights that dynamic soaring enables birds to achieve higher power input and output over shorter time scales.

The fundamental reason for these differences likely lies in the distinct airflow environments utilized by the two soaring types. Static soaring relies on localized updrafts,^6^^,^^28^ which are rare and occur in specific conditions,^4^^,^^28^ such as along mountain ridges. When birds encounter such updrafts, they maximize altitude gain, leading to longer flight cycles. In contrast, dynamic soaring capitalizes on shear flow,^5^^,^^10^^,^^12^ which is more prevalent. Shear flow exists within the atmospheric boundary layer, where proximity to the ground often intensifies it,^5^^,^^24^ providing more energy for birds (Equations 8 and 10). Consequently, dynamic soaring involves frequent short ascents and descents to gain or release energy, leading to a kinetic pattern characterized by immediate energy rise during energy harvest and immediate energy fall during directional flight.

We define this behavior as a “step-selection flight strategy”. Due to the conflict between energy harvest and directional flight, birds segment one cycle into multiple steps, focusing some on energy harvest and others on directional flight. And, this step-selection strategy adapts to environmental conditions. As shown in Figure 4A, at lower wind speeds $\left(W_{\text{ref}}=10\;m/s\right)$, energy harvest and directional flight steps alternate twice within one cycle, as η dominates twice relative to ϵ. Conversely, at higher wind speeds (Figure 4B, $W_{\text{ref}}=20\;m/s$), energy harvest dominates only once, resulting in each phase occurring once per cycle, and the duration of the directional flight phase significantly increases.

### Optimal dynamic soaring trades off energy harvest and directional flight based on shear strength

Bird flight is influenced by both internal and external factors. Energy harvest and directional flight represent birds’ internal flight decisions, while changes in environmental conditions impel birds to adapt their strategies to optimize the balance between the two. Based on Equations 8 and 10, wind shear strength σ signifies a deeper underlying physical significance: it represents the environment’s capacity to provide energy. A larger σ means a greater ability of the environment to supply energy, whereas a smaller σ indicates a weaker ability. For optimal flight or faster arrival at a target, birds prioritize directional flight over energy harvest once they have acquired sufficient energy at higher σ, as illustrated in Figure 4B ($W_{\text{ref}}=20\;m/s$) compared to Figure 4A ($W_{\text{ref}}=10\;m/s$).

**Figure 5:**
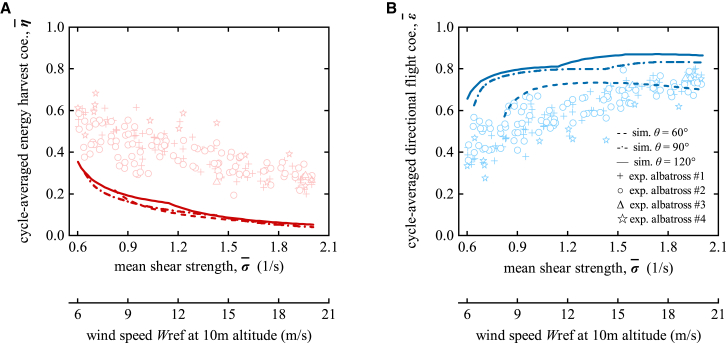
Birds trade off energy harvest ($\bar{\eta }$) and directional flight ($\bar{\epsilon }$) based on the available environmental energy ($\bar{\sigma }$) As $\bar{\sigma }$ increases, $\bar{\eta }$ decreases (A) and $\bar{\epsilon }$ increases (B), indicating that as more energy is available from the environment, birds prioritize directional flight — characterized by flatter and straighter trajectories — over energy harvest, which involves undulating and meandering trajectories. The dashed, dashed-dot, and solid lines represent numerical simulation results for travel directions θ=60°, 90° and 120°. The symbols +, ○, Δ, and ☆ correspond to the data of wandering albatross #1, #2, #3, and #4 from Yonehara et al.’s tracking experiment.^24^ For the experimental data processing method, see Section “data analysis from the literature”. The lower bounds of the numerically optimized solutions for the EOMs are $W_{\text{ref}}=8.2m/s$ for θ=60°, $W_{\text{ref}}=6.4m/s$ for θ=90° and $W_{\text{ref}}=6.0m/s$ for θ=120°, with the upper limit set at $W_{\text{ref}}=20m/s$, based on atmospheric conditions.^24^^,^^25^^,^^30^

Thus, when the external environment offers more energy, birds are more inclined to prioritize directional flight. Conversely, in energy-limited conditions, they engage more in dynamic soaring. The trends of cycle-averaged energy harvest coefficient ($\bar{\eta }$) and directional flight coefficient ($\bar{\epsilon }$) under varying $\bar{\sigma }$ (or, reference wind speed $W_{\text{ref}}$) by simulations are depicted in Figure 5. As $\bar{\sigma }$ increases, $\bar{\eta }$ gradually decreases, indicating that with more available energy, birds can sustain flight with less harvesting. Simultaneously, as $\bar{\sigma }$ rises, $\bar{\epsilon }$ increases, reflecting a preference for rapid directional flight when energy is abundant.

The empirical data from Yonehara et al.'s tracking experiment^24^ is also presented in Figure 5, showing slight individual differences among four albatrosses. For example, Albatross #2 exhibits lower $\bar{\eta }$ and higher $\bar{\epsilon }$ under low wind speed conditions compared to the overall data, while Albatross #4 demonstrates higher $\bar{\eta }$ and lower $\bar{\epsilon }$ than the overall data. These variations suggest that individual differences in animals (e.g., fuel loads, needs, and abilities) contribute to flight performance discrepancies. Nevertheless, the empirical data overall exhibit strong consistency and a clear trend of change. To further analyze the relationships between $\bar{\sigma }$ and $\bar{\eta }$, as well as between $\bar{\sigma }$ and $\bar{\epsilon }$, Generalized Linear Mixed Models (GLMMs)^31^ were applied. Specifically, $\bar{\sigma }$ was included as a fixed effect to assess its overall impact on $\bar{\eta }$ and $\bar{\epsilon }$. The GLMM analysis results reveal a significant negative relationship between $\bar{\sigma }$ and $\bar{\eta }$ (slope=−0.802,p−value<0.001), indicating that as $\bar{\sigma }$ increases, $\bar{\eta }$ decreases. Additionally, a significant positive relationship exists between $\bar{\sigma }$ and $\bar{\epsilon }$ (slope=0.873,p−value<0.001), indicating that as $\bar{\sigma }$ increases, $\bar{\epsilon }$ increases. This observed trend is consistent with the trend predicted by the theoretical simulations, providing empirical support from real experimental observations for the trade-off between energy harvest and directional flight in dynamic soaring.

Also, by comparing the experimental data and numerical data in Figure 5, the experimentally estimated $\bar{\eta }$ is generally higher than the simulated results, whereas the experimental $\bar{\epsilon }$ is lower than predicted by the numerical simulations. This result supports Goto et al.’s^17^ hypothesis that albatrosses adopt a risk-averse strategy in real flight. Our findings indicate that albatrosses prioritize energy harvesting over simply maximizing ${\bar{V}}_{net}$ during actual flight. The reason may be that, unlike theoretical simulations based on steady shear flows, real flight environments involve uncertainties such as turbulence,^32^ gusts, ocean waves,^33^^,^^34^ and precipitation.^4^ These uncertainties may cause birds to deviate from the low-cost envelope of dynamic soaring, increasing their flight burden. To cope with these challenges, birds tend to prioritize energy harvesting to maintain energy reserves, enabling them to confront unpredictable environmental risks and sustain continuous flight in complex and dynamic atmospheric conditions.

The numerical results in Figure 5 show that when $\bar{\sigma }$ is too low ($\bar{\sigma }<0.82\;1/s$ for θ=60°, $\bar{\sigma }<0.64\;1/s$ for θ=90° and $\bar{\sigma }<0.60\;1/s$ for θ=120°), the 3-DOF model fails to generate optimized results. However, experimental data in Figure 5 indicate that birds can still perform dynamic soaring even under these low-wind conditions. Since $\bar{\sigma }$ remains a positive value, the environment theoretically provides energy for birds’ flight (see Equation 10). The model’s failure stems from its exclusive consideration of gliding, neglecting the fact that real birds, such as wandering albatrosses, also engage in flapping flight, spending 1.2–14.5% of their time in slow flapping and 0.1–0.4% in quick flapping.^35^ In low-shear-strength environments, birds use flapping to offset some of the energy required for directional flight or change the direction directly, especially during energy-intensive turning phases.^5^ This ability of birds to alternate between gliding and flapping is also a key feature of the step-selection flight strategy, allowing real-time decisions to optimize their flight state and enhance dynamic soaring.

Given this, we can extend the analysis from gliding-based dynamic soaring to gliding-flapping-mixed and flapping-based dynamic soaring.^1^^,^^10^ Traditionally, flapping is seen as highly energy-intensive, consuming approximately 30 times more energy than gliding,^36^ and thus is thought to be counterproductive for long-distance, low-cost flight. However, flapping plays a proactive role in adjusting energy harvest, expanding the range of environmental conditions from which birds can harvest energy. This is supported by observations of shearwaters, which use wing flapping during transitions from ascent to descent in dynamic soaring.^5^^,^^37^

Therefore, dynamic soaring can be understood from a more generalized perspective: as long as birds harvest energy from spatiotemporal wind gradients, whether through gliding or flapping, the flight qualifies as dynamic soaring. Birds continuously trade off energy harvest and directional flight, irrespective of their flight mode (gliding or flapping) or the strength of the wind shear.

### Conclusion

This paper goes beyond the traditional focus on energy harvest to explore the tradeoff between energy harvest and directional flight in dynamic soaring. When birds perform dynamic soaring toward a target, a conflict arises: maximum energy harvest requires ascending into headwinds or descending with tailwinds in the vertical plane, while fastest directional flight involves flying crosswind in the horizontal plane.

By using a step-selection flight strategy, birds break each flight cycle into smaller steps, prioritizing energy harvest in some phases and directional flight in others. By weaving these steps together, one by one, birds achieve continuous low-cost flight.

Flight decisions during dynamic soaring are shaped by the environmental wind gradient (shear strength), representing the ability of the environment to provide energy and influencing birds’ tradeoff between energy harvest and directional flight. Under weak shear, birds prioritize energy harvest; under strong shear, directional flight takes precedence. Crucially, power flight like flapping may serve as an adaptive response to environmental changes, complementing gliding for sustained energy efficiency.

This research enhances our understanding of avian dynamic soaring under real-world environmental conditions, with potential applications in designing flying robots. The step-selection flight strategy could inform robust machine learning algorithms^38^^,^^39^ for online trajectory planning and control in robotic dynamic soaring, emphasizing the roles of σ (shear strength), η (energy harvest coefficient), and ϵ (directional flight coefficient).

### Limitations of the study

This study primarily focuses on wandering albatrosses, which predominantly utilize gliding-based dynamic soaring,^1^ while the dynamic soaring capabilities of different species require further exploration. For instance, smaller shearwaters typically rely on flapping-based dynamic soaring,^5^ likely due to differences in their aerodynamic characteristics. Understanding the reasons behind these variations could provide valuable insights into species-specific adaptations in dynamic soaring.

Second, the discussion in this paper is centered on crosswind dynamic soaring within the travel direction range of [60°,120°]. Although crosswind dynamic soaring is the dominant mode during dynamic soaring, wandering albatrosses occasionally employ upwind and downwind dynamic soaring as well.^17^^,^^18^^,^^24^^,^^34^ In these flight modes, the relationship between energy harvest and directional flight becomes more complex. For example, in upwind flight, the energy harvest process, which involves flying against the wind, can also be interpreted as movement toward the travel direction. This coupling between energy harvest and directional flight presents a challenge in decoupling and understanding their individual contributions. Further research is needed to explore how energy harvest and directional flight interact in upwind and downwind dynamic soaring.

Third, this study highlights the need for a deeper understanding of dynamic soaring across different scales. According to Goto et al.,^17^ albatross flight can be categorized into three scales: microscale, mesoscale, and macroscale. At the microscale, albatrosses perform dynamic soaring cycles lasting less than 10 seconds, covering tens to hundreds of meters, focusing on immediate aerodynamic interactions with the wind gradient to extract mechanical energy for flight. At the mesoscale, albatrosses cover several kilometers over several minutes, involving a series of soaring cycles. Here, they exhibit more strategic behavior, optimizing travel efficiency by maximizing the distance traveled along the goal direction per unit of energy cost. At the macroscale, albatrosses cover hundreds of kilometers over several hours, engaging in large-scale navigation strategies, such as tacking and increasing the number of turns based on wind and goal directions. In our study, we primarily focus on the microscale to mesoscale range. However, the relationship between energy harvest and directional flight at the mesoscale and macroscale remains an open question. For instance, at the macroscale, birds may prioritize foraging, raising the possibility of introducing a new variable, such as a “flight coverage coefficient”, to quantify their foraging efficiency. These large-scale trade-offs warrant further investigation to better understand the dynamic soaring across different spatial and temporal scales.

Fourth, as one of the two major soaring strategies, static soaring, which primarily harvests energy from vertical updrafts, requires further investigation. The trade-off in static soaring could be even more pronounced, as energy gain predominantly occurs in the vertical direction, while directional flight proceeds horizontally. This conflict likely necessitates a delicate tradeoff for birds engaged in static soaring, offering potential insights into the flight and migratory behaviors, such as frigatebirds^4^ and raptors.^28^

Additionally, while this paper presents a framework for understanding the trade-off between energy harvest and directional flight in dynamic soaring, the proposed concepts require further validation through rigorous experimental studies, particularly the collection of altitude data during bird flight, or advanced numerical and theoretical analyses, such as incorporating wind models with fluctuating wind speeds. These efforts are essential to confirm the applicability and robustness of the findings across diverse environmental conditions. Furthermore, such research could provide valuable insights for the design of flight strategies in UAVs, offering practical applications in engineering and robotics.

## Resource availability

### Lead contact

Further information and requests for resources and reagents should be directed to and will be fulfilled by the lead contact, Yang Xiang (xiangyang@sjtu.edu.cn).

### Materials availability

This work did not generate new unique reagents and components.

### Data and code availability

• All raw data reported in this paper will be shared by the lead contact upon request.
• All custom-made scripts and codes for analysis are available for request by contacting the lead author, Yang Xiang (xiangyang@sjtu.edu.cn).
• Any additional information required to reanalyze the data reported in this paper is available from the lead contact upon request.

## Acknowledgments

The authors express their gratitude to Zhuoqi Li, Yimin Wu, and Wenchang Wang for their logical discussions and wishes to acknowledge the authors of ref. 24, namely Y. Yonehara, Y. Goto, K. Yoda, Y. Watanuki, L.C. Young, H. Weimerskirch, C.A. Bost. and K. Sato for their enabling work in publishing the characteristics of wandering albatross flights. This work was supported by the National Natural Science Foundation of China (NSFC) under Grant Nos. 12202273 and 91952302, the China Postdoctoral Science Foundation (Grant No. 2018M642007), and Shanghai Jiao Tong University’s “Double First-Class” Project.

## Author contributions

Conceptualization, L.C. and Y.X.; methodology, L.C. and Y.Y.; investigation, L.C. and Y.Y.; writing–original draft, L.C.; writing–review and editing, L.C., Y.Y., and Y.X.; funding acquisition, Y.X. and S.Q.; resources, Y.X. and S.Q.; supervision, Y.X. and H.L.

## Declaration of interests

The authors declare no competing interests.

## Declaration of generative AI and AI-assisted technologies in the writing process

During the preparation of this work the authors used ChatGPT-4o to improve language. After using this tool/service, the authors reviewed and edited the content as needed and takes full responsibility for the content of the publication.

## STAR★Methods

### Key resources table

**Table:** 

REAGENT or RESOURCE	SOURCE	IDENTIFIER
**Deposited data**		

Raw data and code	This paper	N/A
Experimental flight data of albatrosses	https://doi.org/10.5061/dryad.3pb86	Yonehara et al.^24^

**Software and algorithms**		

Python (version 3.8.7)	https://www.python.org/	The Python Software Foundation
IPOPT (version 1.0)	https://coin-or.github.io/Ipopt/	N/A

### Experimental model and study participant details

The experimental model and study participant details are based on a combination of numerical simulations and empirical flight data from wandering albatrosses (*Diomedea exulans*). The numerical framework employed a 3-degree-of-freedom (3-DOF) point-mass glider model operating in the logarithmic/sigmoid shear wind profiles. The empirical flight data analysis included flight trajectories of four individual albatrosses (Albatross #1–#4) engaged in prey-capture events, with data sourced from Yonehara et al.^24^ This dataset comprised 185 hours of high-resolution latitude and longitude recordings sampled at 1 Hz, providing insights into long-distance foraging behavior.

### Method details

#### Constraints of the optimization problem

The constraints consist of five main components: continuous-time constraints, boundary constraints, direction constraints, physical constraints, and technical constraints, as summarized in Table S2 in the supplemental information. The continuous-time constraints are discretized using a collocation method, while the boundary, direction, physical, and technical constraints are applied to maintain realistic and consistent flight behavior.

The continuous-time constraints are formulated using a finite-dimensional optimization approach based on direct collocation. The time interval over one period $\left[0,n_{1}T,n_{2}T,\ldots ,n_{N-1}T,T\right]$ where $0<n_{1}<\cdots <n_{N-1}<1$, and the spacing between steps need not be uniform. For clarity, the shorthand notation $s_{i}\hat{=}s\left(n_{i}T\right)$ and $u_{i}\hat{=}u\left(n_{i}T\right)$ are used. Following the work of Bousquet et al.^16^ and Bower,^18^ the continuous-time constraints,

$$s\left(n_{i}T\right)=\int_{n_{i-1}T}^{n_{i}T}f\left(s\left(t\right),u\left(t\right)\right)dt\text{,}$$

can be approximated by,

$$s_{i}=s_{i-1}+\frac{1}{6}\left(f\left(s_{i},u_{i}\right)+4f\left(s_{m_{i}},u_{m_{i}}\right)+f\left(s_{i-1},u_{i-1}\right)\right)\left(n_{i}-n_{i-1}\right)T\text{,}$$

where,

$$s_{m_{i}}=\frac{1}{2}\left(s_{i}+s_{i-1}\right)-\frac{1}{8}\left(f\left(s_{i},u_{i}\right)-f\left(s_{i-1},u_{i-1}\right)\right)\left(n_{i}-n_{i-1}\right)T\text{,}$$

And

$$u_{m_{i}}=\frac{1}{2}\left(u_{i}+u_{i-1}\right)\text{.}$$

The discrete form of the continuous-time constraints is thus expressed as,

$$C_{i}=s_{i}-s_{i-1}-\frac{1}{6}\left(f\left(s_{i},u_{i}\right)+4f\left(s_{m_{i}},u_{m_{i}}\right)+f\left(s_{i-1},u_{i-1}\right)\right)\left(n_{i}-n_{i-1}\right)T=0\text{.}$$

The boundary constraints ensure continuity between different dynamic soaring cycles and are defined as U(0)=U(T),ψ(0)=ψ(T),γ(0)=γ(T),z(0)=z(T). The direction constraint is specified by tanθ=Δx/Δy, where $\Delta x=x_{N}-x_{0}$ and $\Delta y=y_{N}-y_{0}$ represent the distances between the end and start points in the x and y directions, respectively. Physical constraints include the maximum lift coefficient of 1.5, the load factor n less than 3, and the flight altitude greater than 0.5m, as referenced from Goto et al.^17^ Technical constraints involve limitations on the angles ϕ, ψ and γ to facilitate numerical computations.

#### Model validation

The model was validated by comparing its results with those of Sachs' study,^22^ which have been widely confirmed as accurate by numerous researchers.^16^^,^^18^ To ensure consistency with Sachs' study, three adjustments were made to the model constraints in the main text:

(1) The optimization objective was changed to minimize the reference wind speed ($W_{\text{ref}}$);
(2) The minimum vertical displacement constraint was set to z≥1.5m;
(3) The constraint on travel direction was removed, meaning no predefined target direction was imposed on the bird’s flight.

Under these conditions, the optimized results were compared with Sachs' results, as shown in Figure S1 in the supplemental information. The comparison of flight trajectories in the x, y, and z directions demonstrates a high degree of agreement, confirming the accuracy of our algorithm.

Furthermore, the minimum wind speed obtained under these conditions was compared. Our model yielded a minimum wind speed of 8.57m/s with a cycle period of 7.00s, closely matching 8.6m/s and 6.6s as the results of Sachs.^22^ This validation confirms the accuracy of our algorithm. It is also important to note that the minimum wind speed calculated in the main text is approximately 6m/s, or more precisely, 5.81m/s, because the main text uses a constraint of z≥0.5m.

In summary, when the constraints are consistent with those of previous studies, our model produces results that align with established findings, thereby validating the correctness of the model.

#### Influence of Sigmoid wind model

The purpose of this section is to validate the applicability of the study’s conclusions under the sigmoid wind model, thereby enhancing the robustness of the results. Since albatrosses often fly very close to the ocean surface, the validation under the sigmoid wind model serves as a supplement to the logarithmic wind model used in the main text.

Common wind profiles include the logarithmic model, linear model, and sigmoid model.^9^ The logarithmic model is widely used for simulating atmospheric boundary layers. The linear model is primarily applied when the wind speed tends to follow a linear distribution at higher altitudes (i.e., the log function approximates a linear distribution at greater heights), and its simplicity makes it popular for use. The sigmoid model focuses on velocity discontinuities near the surface, often used to model wind speed transitions in wave or mountain lee areas, where weaker winds exist. The main difference between the sigmoid and logarithmic models is that the sigmoid model can maintain near-zero or low wind speeds over a certain height range such as the zero/low-wind-speed region behind the wave, while the logarithmic model’s wind speed curve only approximates zero over a narrow range, making the sigmoid model more suitable for simulating wave and mountain flow conditions.

The math expression of the sigmoid wind model is,

$$W\left(z\right)=\frac{W_{\text{ref}}}{1+\exp\left(-\frac{z}{h_{\text{ref}}}\right)}$$

where, $W_{\text{ref}}$ is the reference wind speed and $h_{\text{ref}}$ is the reference height. A comparison of the sigmoid and logarithmic wind models is shown in Figure S2. As illustrated, the sigmoid wind model exhibits a faster increase in wind speed and a steeper gradient at lower altitudes, which has potential implications for analyzing dynamic soaring near the ocean surface.

The trajectories and basic variable for three travel directions (θ=78°, 90°, and 120°) are shown in Figure S3. Under the conditions of the sigmoid wind model at $h_{\text{ref}}=1.0m$ and $W_{\text{ref}}=10m/s$, the trajectory for θ<78° essentially consists of two dynamic soaring cycles and one-cycle flight cannot be performed. Thus, according to the premise of the paper, which focuses on one-cycle microscale dynamic soaring, the results for θ∈[60°,78°) showing two dynamic soaring cycles, are excluded from further analysis. As presented in Figure S3, under the sigmoid wind model, dynamic soaring also occurs through climbing-diving cycles to trade energy for directional flight. During energy rise phases, birds harvest energy from the environment, while in energy fall phases, they allocate it to maintain directional flight. The associated physical quantities are consistent with those in Figure 2 of the main text.

Additionally, the changes in the energy harvest coefficient (η) and directional flight coefficient (ϵ) over one cycle are presented in Figure S4. The step-selection flight strategy for dynamic soaring is also observed under the sigmoid wind model. By comparing Figures S4A and S4B, it is evident that under higher wind speeds (more energy available), η is generally smaller, while ϵ is larger. This supports the conclusion that as more energy is available from the environment, birds prioritize directional flight over energy harvest.

In summary, the results presented in this section demonstrate that the core conclusions regarding the trade-off between energy harvest and directional flight remain consistent under sigmoid wind model, indicating the robustness of findings.

### Quantification and statistical analysis

Generalized Linear Mixed Models (GLMMs)^31^ were applied to analyze the relationship between shear strength (σ) and flight performance metrics (energy harvest coefficient η and directional flight coefficient ε). Results confirmed a significant negative correlation between σ and η (p<0.001) and a positive correlation between σ and ε (p<0.001), supporting the trade-off hypothesis.

The study’s reliance on 2D tracking data necessitated approximations for pitch angle (γ), potentially introducing errors. Individual variability among albatrosses (e.g., Albatross #2’s preference for directional flight vs. #4’s focus on energy harvest) highlighted behavioral differences not fully captured by the model. Future work could benefit from high-resolution 3D tracking to refine kinematic estimates.
