# Supplementary material for: Optimal dynamic soaring trades off energy harvest and directional flight
Source: iScience. 2025 Apr 28;28(6):112540. doi: 10.1016/j.isci.2025.112540 (PMC12141101; doi:10.1016/j.isci.2025.112540)
Supplement: Document S1. Figures S1–S5 and Tables S1 and S2 [file mmc1.pdf]

iScience, Volume *28*

Supplemental information

Optimal dynamic soaring trades off energy harvest and directional flight

Lunbing Chen, Yufei Yin, Yang Xiang, Suyang Qin, and Hong Liu

S.1. Basic properties for modelling albatross

Tab.S 1 Basic properties of modelling albatross. In addition to the variables already introduced in the main text, $n_{\max}$ represents the maximum load factor, where $n = L/mg$. Related to sub-Section "Modelling of dynamic soaring" in the main text.

Parameter	m (kg)	S (m^2)	λ	C_{D0}	$n_{\max}$
Value	8.5	0.65	16.81	0.033	3

S.2. Constraints of the optimization problem

Tab.S 2 Constraints applied in the numerical optimization. Related to sub-Section "Constraints of the Optimization Problem" in the main text.

#	Type	Expression
1	continuous-time constraints	$C_i = 0,\ i = 1, \dots, N$
2	boundary constraint	$(U_N, \psi_N, \gamma_N, z_N, x_N) = (U_0, \psi_0, \gamma_0, z_0, x_0)$
3	direction constraint	$\tan\theta = \Delta x/\Delta y$
4	physical constraints	$-0.2 \le C_{L,i} \le 1.5$
		$n_{\max} \le 3$
		$z_i \ge 0.5$ m
5	technical constraints	$-8/9 \times \pi/2 \le \phi_i \le 8/9 \times \pi/2$
		$-\pi \le \psi_i \le \pi$
		$-\pi/2 \le \gamma_i \le \pi/2$

S.3. Model validation

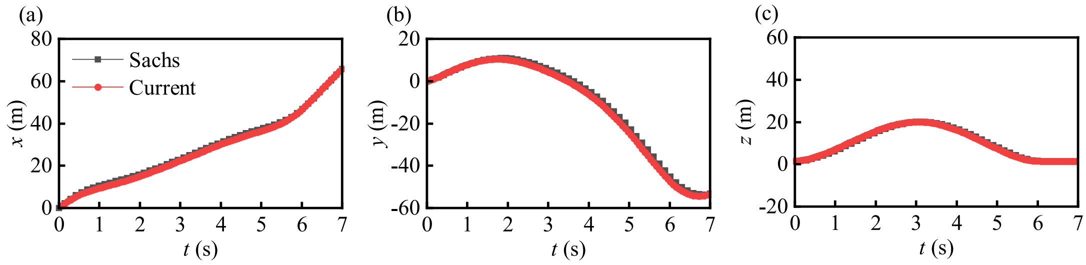

Fig.S 1 Simulation results of this study align with Sachs' results when the optimization objective is set to minimize the reference wind speed W_{ref} and $\mathrm{z} \geq 1.5\ \mathrm{m}$. Related to sub-Section "Model Validation" in the main text.

S.4. Influence of sigmoid wind model

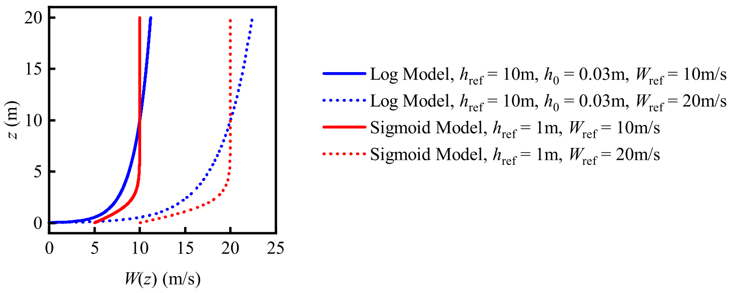

Fig.S 2 Wind profile comparison between sigmoid model and logarithmic model. Related to sub-Section "Influence of Sigmoid Wind Model" in the main text.

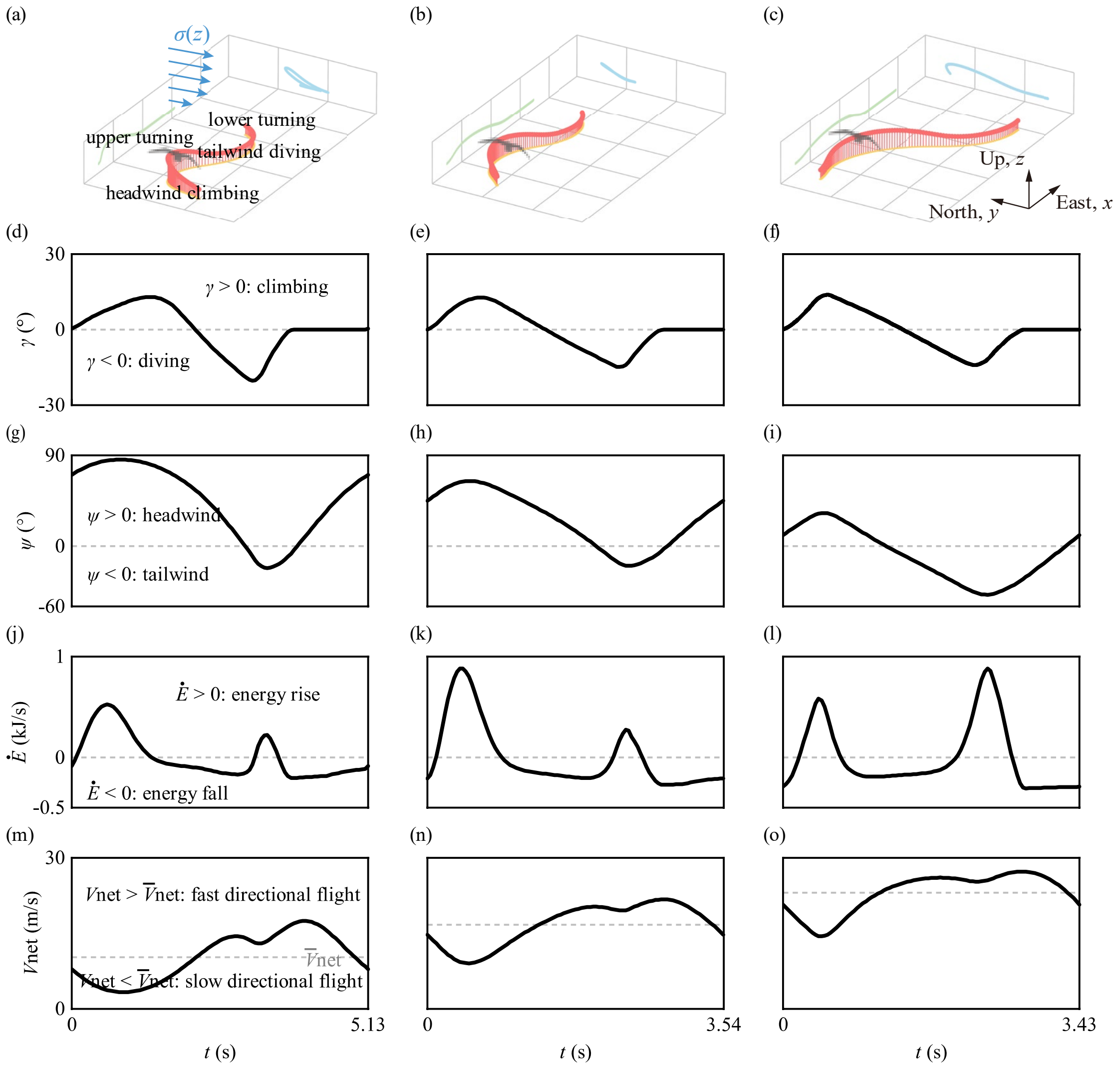

Fig.S 3 Dynamic soaring trajectories (a-c) and basic physical variables (d-o) under sigmoid wind model at the $h_{\mathrm{ref}} = 1.0\mathrm{m}$ and $W_{\mathrm{ref}} = 10\mathrm{m/s}$. The three columns of the sub-figures correspond to the results for $\theta = 60°$, $90°$, and $120°$ respectively. Related to sub-Section “Influence of Sigmoid Wind Model” and Fig.2 in the main text.

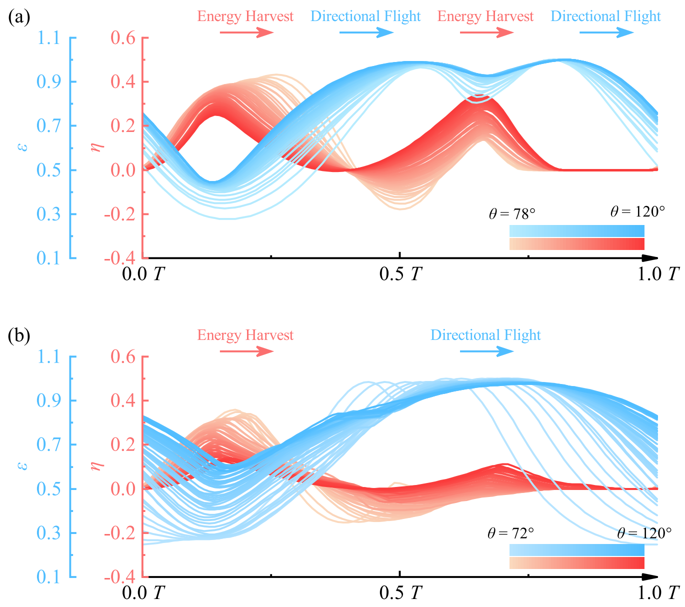

Fig.S 4 Energy harvest coefficient η (light red) and directional flight coefficient ϵ (light blue) change with time in one cycle under the sigmoid wind model. (a) For $h_{\mathrm{ref}} = 1.0\mathrm{m}$ and $W_{\mathrm{ref}} = 10\ \mathrm{m/s}$; (b) For $h_{\mathrm{ref}} = 1.0\mathrm{m}$ and $W_{\mathrm{ref}} = 20\ \mathrm{m/s}$. Related to sub-Section "Influence of Sigmoid Wind Model" and Fig.4 in the main text.

S.5. Supplementary results of airspeed and lift coefficient

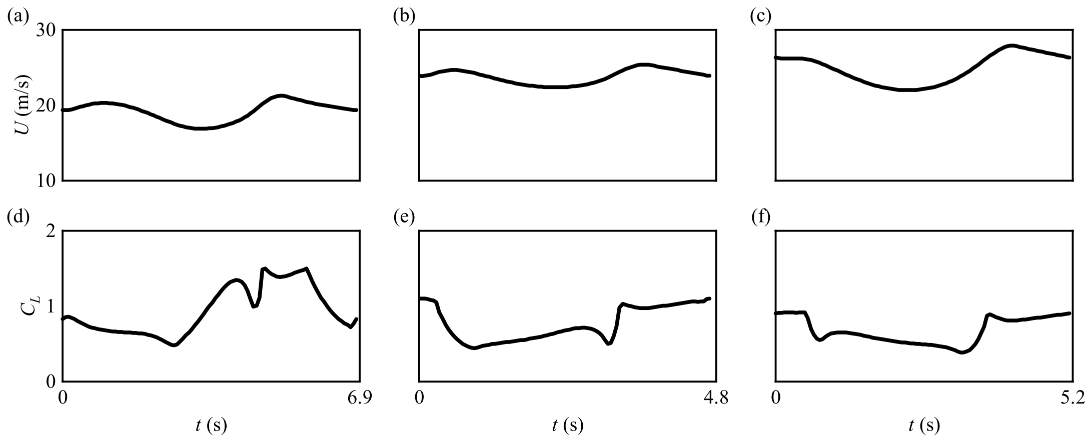

Fig.S 5 Supplementary results of airspeed U (a-c) and lift coefficient C_L (d-f). The first to third columns of figures correspond to flight directions $\theta = 60°$, $90°$, and $120°$. Simulations are performed with the objective of maximizing $\bar{V}_{\mathrm{net}}$ at a reference wind speed (W_{ref}) of $10\ \mathrm{m/s}$. Related to Fig.2 in the main text.
